# Supplementary material for: Are carbon emissions trading and green financial instruments synergistic? -Comprehensive quantitative research based on content analysis
Source: PLoS One. 2024 Mar 7;19(3):e0298601. doi: 10.1371/journal.pone.0298601 (PMC10919601; doi:10.1371/journal.pone.0298601)
Supplement: S1 Data — (ZIP) [file pone.0298601.s001.zip › Data/地级市Z_F工作报告.zip!/─┌├╔╣┼/║⌠┬╫▒┤╢√╩╨/└┤╫╘╣1⁄2╓┌║┼í╛┬φ┐╦╩2╛▌═°í┐_2017.docx]

各位代表：

现在，我代表呼伦贝尔市人民政府向大会报告工作，请予审议，并请市政协委员和列席会议的同志提出意见。

一、2016年工作回顾

过去的一年，是我市发展进程中不平凡的一年。面对经济下行的严峻挑战和有气象记录以来最为严重的旱灾影响，在自治区党委、政府和市委的坚强领导下，市政府团结带领全市各族人民，统筹稳增长、促改革、调结构、防风险、惠民生，保持了经济平稳发展、社会繁荣进步、各民族团结奋进的良好局面。

（一）综合实力稳步提升。地区生产总值完成1620.9亿元,增长7%。一般公共预算收入完成106亿元，按可比口径增长10.6%。固定资产投资完成982亿元，增长6.1%。社会消费品零售总额完成600亿元，增长10%。城镇居民人均可支配收入完成28885元，增长7.6%。农村牧区人均可支配收入完成12540元，增长7.8%。174个市本级重点项目进展顺利，完成投资486.6亿元。大灾之年，粮食产量仍然达到120.7亿斤，居全区第二位。牧业年度牲畜存栏达到2176.3万头（只）。财政投入资金10亿元，用于稳工业和扶持中小企业，稳增长成效明显。金融支持实体经济作用显著，社会融资总规模达到1875.2亿元，增长15.4%，信贷结构不断优化，贷款增速达到16.8%，高于存款增速0.6个百分点。全市招商推介会签约项目66个，签约额超过1000亿元。成功举办了冬季那达慕、全域旅游冬季高峰研讨会和海拉尔国际音乐名城高峰研讨会。满洲里中俄边境旅游区晋升为国家5A级景区。扎兰屯市和鄂温克旗分别被环保部授予“国家级生态市”和“国家级生态旗”荣誉称号。“呼伦贝尔物产”系列广告在央视展播，呼伦贝尔肉业集团“中荣及图”商标荣获中国驰名商标。呼伦贝尔蒙拓农机科技股份有限公司等3家企业成功在新三板挂牌。我市的综合经济实力和地区影响力得到进一步提升。

（二）结构调整持续优化。三次产业结构由16.5:44.6:38.9优化为15.3:44.7:40。农区牲畜头数占全市牲畜总数比重由70.5%提高到72%，畜牧业产值占第一产业比重由35%提高到37%，粮经饲种植比例由87：10：3调整为83.4:14.5:2.1。非资源型产业实现产值870亿元，占工业总产值的67.8%，增速高于资源型产业8.3个百分点。第三产业对经济增长贡献率由42.5%提高到55.5%。旅游收入完成528亿元，增长17.7%；全年接待游客1553.6万人次，增长9.6%。电子商务迅猛发展，线上交易额达到11.5亿元。快递业快速发展，全年业务量近3200万件，增长187.5%。非公经济增加值占地区生产总值比重由53.6%提高到54.1%。“三去一降一补”扎实推进,完成了75万吨煤炭和7万吨钢铁去产能任务，消化商品房库存138.3万平方米，直供电交易降低企业成本超过1.2亿元。

（三）生态保护全面加强。严格落实国有林区天然林商业性停伐政策。绿化造林78.6万亩。红花尔基12.5万亩退化土地碳汇造林项目在国家发改委备案，填补了自治区在林业领域开发减排项目的空白。设立了呼伦贝尔林业生态发展基金，为国家储备林、林业生态工程和林业基础设施建设搭建了融资平台。认真落实新一轮草原生态保护补助奖励政策，发放补奖资金6.3亿元。退牧还草190万亩。扎实开展打击毁林毁草专项行动，退耕33.6万亩。《内蒙古自治区呼伦湖国家级自然保护区条例》颁布实施，呼伦湖生态环境综合治理一期系列工程全面推进。自然资源资产负债表编制试点和水生态文明试点年度工作如期完成。大气、水、土壤污染防治工作取得积极进展。完成了年度节能节水减排等约束性指标任务。在接受国务院第三次大督查、中央环保督察和领导干部自然资源资产离任审计过程中，我们积极配合、直面问题、认真整改，及时回应社会关切，依法对相关责任人和责任主体进行了严肃查处。

（四）城乡面貌明显改善。住建领域完成投资240亿元，其中市政基础设施项目完成投资55亿元。基本完成“十个全覆盖”既定任务，当年完成投资120.7亿元，农区、牧区、林区、垦区生产生活整体环境发生显著变化。综合交通体系建设完成投资121.7亿元，扎兰屯—阿荣旗高速公路等12个重点项目进展顺利，满洲里—阿拉坦额莫勒、阿拉坦额莫勒—阿木古郎一级一幅公路实现通车。滨洲铁路电气化改造主体完工，齐齐哈尔—海拉尔—满洲里客运专线项目正式列入国家中长期铁路发展规划。海拉尔东山机场扩建主体工程竣工，扎兰屯支线机场实现通航，新右旗、阿荣旗、莫旗、陈旗和满归通用机场建设有序推进。水利建设完成投资18亿元，扎敦水利枢纽大坝合龙，Z866项目临时工程开工建设,尼尔基水利枢纽下游内蒙古30万亩灌区等工程扎实推进。电网建设完成投资21亿元，重点实施了海北—牙克石—扎兰屯500千伏输变电工程等35个110千伏及以上电网工程建设项目。

（五）改革开放不断深入。完成了78项经济、生态、社会和文化领域改革任务。承接了以“两供一业”为主的大兴安岭国有林区社会管理和公共服务职能。地方国有林场“三供一业”职能基本剥离。全面推行“双随机一公开”监管，市直部门全部编制了责任清单。完成了“五证合一、一照一码”、不动产统一登记等制度改革。新预算法得到落实，以“营改增”为主的财税体制改革全面推开。土地和草牧场确权登记颁证工作稳步推进。完成了阿荣旗全国农村改革试验区和农村承包土地经营权抵押贷款试点年度任务。人才发展体制机制改革逐步深化。积极培育市场主体，成立了呼伦贝尔文化旅游投资（集团）公司、呼伦湖生态环境保护与开发投资公司、呼伦贝尔交通投资公司。积极融入国家“一带一路”和新一轮东北振兴战略，中俄蒙合作先导区和中蒙跨境经济合作区前期工作有序推进，满洲里综合保税区实现封关运营，扎赉诺尔区被国家列为新增4个陆地边境县之一。海拉尔·中俄蒙博览会晋升为国家级展会。跨境货运专列达到846列，增长40.1%，货值116亿元，增长19.6%。全市口岸进出境货运量完成3155万吨，外贸进出口总额完成27亿美元。全年引进国内（市外）资金652亿元。

（六）民生工作力度加大。公共财政支出继续向民生领域倾斜，支出达309.5亿元，增长11%，占公共财政支出比重达74.6%。精准扶贫成效明显，投入各类扶贫资金30.3亿元，精准脱贫24960人。开工各类保障性安居工程69434套，建成29363套。发放各类农业补贴35亿元，有效促进了农民直接增收。城镇新增就业33942人，农牧民转移就业76781人，高校毕业生就业10595人，零就业家庭实现至少1人就业。大众创业、万众创新良好氛围初步形成，呼伦贝尔市创业园众创空间入选第三批国家级众创空间名单。发放购煤补助1.2亿元、助学金9866万元。建立了统一的城乡居民基本医疗保险制度。企业退休人员养老金人均同比月提高151元。城镇最低生活保障标准同比月提高20元，农村牧区最低生活保障标准同比年提高200元。“菜篮子”工程扎实推进，新增蔬菜种植面积1.5万亩，达到19.5万亩。海拉尔—鄂温克巴彦托海镇公交线路如期开通，中心城区公交一体化改革取得实质性进展，市民出行条件得到改善。

（七）社会事业稳步发展。科技成果转化应用水平逐步提高，产学研合作步伐加快，成立了呼伦贝尔农垦草牧业等7个院士专家工作站，成功举办了全区科技创新驱动现代农牧业发展现场会和全市科技创新大会，出台了《呼伦贝尔市人民政府关于促进科技创新若干意见》。各级各类教育协调发展，满洲里市、额尔古纳市、牙克石市和扎赉诺尔区通过了国家义务教育发展基本均衡县评估认定。公共文化服务体系建设进展良好，文化产业逐步发展壮大。医药卫生体制改革稳步推进，基本医疗和公共卫生服务能力进一步加强，呼伦贝尔市人民医院晋升为三甲医院。自治区第九届少数民族传统体育运动会筹备工作、第十四届全国冬运会场馆建设及改造有序推进。新闻出版广电、国资监管、档案史志、外事侨务、人事、规划、气象、人防、地震、工青妇、红十字、残联、文联、科协、关工委和老龄等工作均有新进展。

（八）民主法治持续推进。认真执行市人大及其常委会决定，自觉接受市政协民主监督，广泛听取各民主党派、工商联和无党派人士意见。全年办理人大代表议案1件、建议126件、政协委员提案193件，办复率100%。扎实开展“两学一做”学习教育，政府系统工作作风持续改进。政务公开工作质量和实效同步提高。行政执法和公正司法水平得到提升。行政监察、审计监督和社会监督作用得到充分发挥。全国文明城市创建工作正式启动，精神文明建设迈出新步伐。认真落实民族宗教政策，社会主义新型民族关系继续巩固。落实“四个清单四个清”信访机制，解决了430个突出问题。执行“四个最严”要求，食品药品安全监管工作得到加强。安全生产事故起数和死亡人数实现双降。241个城镇社区办公、活动场所面积达到300平方米以上，达标率93%。社会治安大局保持稳定，立体化社会治安防控体系不断完善。“双拥”和边防工作得到加强。人民群众安全感和满意度进一步提高。

（九）抗旱减灾取得实效。面对重大旱灾，在国家、自治区亲切关怀和大力支持下，全市上下积极应对、科学施策。通过下拨9亿元救灾资金、投入1.2万台套抗旱设备、开展人工增雨等多项措施缓解了旱情，把农业损失降到了最低限度。多方调草69万吨，基本解决了牧区牲畜过冬问题。通过购买爱心羊、实施价格补贴等措施，引导牧民加大牲畜出栏力度，最大限度减少牧民损失，有效保护了农牧民利益。

各位代表，过去一年的成绩来之不易。这是自治区党委、政府和市委正确领导的结果，是市人大、政协有效监督的结果，是全市各族人民团结奋斗的结果，也是社会各界鼎力支持的结果。在此，我代表呼伦贝尔市人民政府，向全市各族人民，向人大代表、政协委员和各民主党派、人民团体，向驻呼军警部队指战员和所有关心支持呼伦贝尔改革、发展、建设的同志们、朋友们，表示衷心的感谢!

在看到成绩的同时，我们也要正视发展中存在的困难和问题：一是经济下行压力持续，稳定经济增长、守住发展底线任务艰巨。二是工业经济下滑幅度较大，部分行业、企业经营困难。三是各类投资主体投资欲望不强，项目落地面临诸多制约因素。四是产业结构不合理，发展方式不优，调结构转方式任重道远。五是环保督察暴露问题较多，守住生态底线的措施还不够到位。六是城乡发展不均衡，基础设施建设依然滞后。七是财政刚性支出增加，新的税源尚待培育，财政收支矛盾加剧。八是脱贫攻坚压力加大，守住民生底线任务较重。九是部分干部责任意识、担当意识不强，工作作风亟待改进。这些问题，我们将高度重视，采取有效措施，切实加以解决。

二、2017年工作总体安排

今年是全市经济社会发展进程中特别重要的一年，做好政府工作，必须全面贯彻落实党的十八大、十八届三中、四中、五中、六中全会精神以及习近平总书记系列重要讲话和考察内蒙古重要讲话精神，全面贯彻落实自治区第十次党代会、自治区党委十届二次全会精神，全面贯彻落实市第四次党代会和市委四届二次全会暨全市经济工作会议精神，紧紧围绕“两区三地一家园”目标定位，牢固树立和践行新发展理念，促进新型工业化、信息化、城镇化、农牧业现代化、绿色化协同发展，坚持稳中求进工作总基调，坚决守住发展、生态和民生三条底线，向改革开放要活力，向创新发展要动力，真抓实干，攻坚克难，实现美丽与发展双赢，以优异的成绩，向党的十九大和自治区成立70周年献礼！

主要预期目标是：地区生产总值增长7.5%左右，固定资产投资增长10%，一般公共预算收入按可比口径增长7.5%，社会消费品零售总额增长10%，城乡居民人均可支配收入分别增长8%和8.5%。完成自治区下达的各项约束性指标任务。

实现上述预期目标，必须抓好以下九个方面的工作：

（一）以绿色化为引领，全面加强生态建设

推进生态保护与建设。紧紧围绕打造全国可持续发展的生态环境建设保护示范区目标，继续实施天然林保护工程，做好国家储备林基地建设，巩固天然林商业性停伐成果。绿化造林75万亩。落实新一轮草原生态保护补助奖励政策，全年禁牧1687万亩、草畜平衡8671万亩。退牧还草122万亩。按照国家耕地、草原、河湖休养生息规划要求，实施“养、退、休、轮、控”举措，有计划、分步骤还林还草还湿地。加强水环境综合治理，完成呼伦湖生态环境综合治理一期系列工程，同步推进贝尔湖等湖泊和域内水系生态修复及管护。继续实施沙区综合治理，巩固治理成果。

加强生态环境整治。严格控制开发强度，在项目准入上，坚决做到不符合主体功能区规划的项目不上、不符合环保要求的项目不上、不符合产业政策的项目不上。加强大气、水、土壤污染防治。推动燃煤锅炉脱硫、脱硝、除尘设施升级和超低排放改造，淘汰10蒸吨及以下老旧锅炉。确保各旗市区生活污水处理厂和各工业园区污水处理厂稳定运行、达标排放。重点做好工业、农牧业面源污染和矿山地质环境综合治理。以“钉钉子”的精神抓好中央环保督察问题整改落实工作，以零容忍的态度严厉打击环境违法行为。

加快生态文明制度建设。严格落实国家主体功能区规划，严守各类生态红线，全力推进水生态文明试点市各项工作。实施最严格的水资源管理制度，制定《呼伦贝尔市地下水管理办法》，加强饮用水水源地保护。继续做好自然资源资产负债表编制和领导干部自然资源资产离任审计试点工作。深化国有林区改革，完成地方国有林场改革。积极培育生态文化，倡导绿色低碳生活方式。

（二）以供给侧结构性改革为主线，全力以赴稳增长

扎实推进“三去一降一补”。科学合理去产能，在确保稳定的前提下，坚决退出不达标、不环保、能耗高、不安全的落后产能，年内淘汰煤炭产能30万吨。因城施策去库存，积极打通去库存和棚户区改造的结合通道，消化库存。防范风险去杠杆，继续优化信贷结构，加快各类投融资平台建设，严格规范政府举债行为。多措并举降成本，争取电力同网同价、推进电力多边交易降低电价，加快生产性物流配套建设，继续开展涉企收费管理和体制机制创新，想方设法降低企业生产成本。突出重点补短板，集中力量，加快进度，着力解决基础设施、农田水利、口岸建设等瓶颈制约。

加大投资力度，守住发展底线。把资金重点投向绿色农畜林产品加工、全域旅游、公共服务等国家重点支持的发展领域，提高投资的有效性和精准性。全年初步安排市本级重点项目211个，总投资1495亿元，当年完成投资518亿元。发挥好财政资金的撬动作用，充分利用各种金融工具和平台，进一步拓宽投融资渠道，不断优化金融资源配置，保证重大项目融资需求。全面落实国家和自治区支持中小微企业发展的各项政策，完善中小微企业融资、担保、创业和服务平台，充分发挥非公企业的作用，研究破解制约民间投资的突出问题，释放本土企业能量，激发民间投资热情。

加大项目谋划和招商引资力度。按照先谋划项目后精准招商的思路，加强与东北老工业基地、环渤海、长三角、珠三角等地区的密切联系，开展针对性、实效性招商，形成建成一批、开工一批、储备一批的梯次格局。深化“亲商”、“清白”的新型政商关系，全面履行政府营造环境的主体责任，打造高效率、无障碍、开放式的发展环境。

（三）以调存量优增量为关键，稳住工业经济

加快传统产业转型升级。强化科技支撑，鼓励技术创新，以高标准的环保要求作为企业生存发展的前提，对现有的煤电化、金属冶炼等能源消耗高、资源利用率低、污染减排压力大的企业进行技术改造，重点督促北方药业等环保不达标企业尽早达标，恢复生产。加大对乳业、建材等产业的整合重组力度。盘活存量工业，对完成一定工程量和具备条件的项目，积极帮助企业解决问题，使其尽快达产达效。

壮大新兴产业规模。积极发展装配式现代木结构建筑产业，支持满洲里联众木业、根河根林木业等企业提升档次、扩大规模、加快发展。鼓励玉米转化、牧机制造、新能源等市场前景好的产业扩大产能、延伸产业链条、提高产品附加值。启动阜丰公司10万吨苏氨酸和10万吨赖氨酸项目前期工作。鼓励华德、瑞丰、蒙拓等牧机制造企业提高数字化、智能化水平。推动光伏发电“领跑者”示范基地规划纳入国家实施方案。引导企业运用互联网思维和信息技术谋划产业发展，发挥“互联网+”的乘数效应。推动海拉尔华为大数据中心、中网科技云计算中心二期项目前期工作。

加快工业园区建设。调整优化园区产业布局。鼓励支持各工业园区继续完善配套设施，提升污水处理、集中供热和铁路专用线等基础设施保障能力，为入园企业发展提供全方位、全天候、全过程的配套保障。科学谋划园区招商和承接产业转移工作，提高园区产业关联度，形成上下游相衔接、资源循环利用的集群化、规模化发展态势。

（四）以培育名优特精为方向，加速农牧林产业化

提升综合生产能力。全年粮食产量稳定在120亿斤以上，牧业年度牲畜存栏达到2200万头（只），肉类、牛奶产量稳定在25万吨和120万吨以上。分区、分类、分品种做好农牧业产业发展规划，优化农牧业区域布局。玉米种植面积稳定在850万亩，大豆种植面积扩大到850万亩。新增高效节水灌溉农田50万亩、高标准农田65万亩。实施土壤健康行动20万亩，打造绿色有机无公害基地。扩大肉牛、肉羊和特色养殖等基地规模。积极推动岭东地区中高端设施蔬菜基地建设。

提高产业化水平。全面落实农业供给侧结构性改革，紧紧围绕市场需求，以增加农牧民收入、保障有效供给为目标，突出优质优价，打造地方特色物产品牌，全链条提升供给质量和效益，推动一二三产业融合发展。支持农垦集团在市场营销、产品升级和品牌建设上取得突破，培育壮大健康食品产业，发挥好产业化航母的示范带动作用。通过招商引资、整合现有企业、组建新的投融资公司等途径，形成一批新的龙头企业。推进耕地草场、机械设备、牲畜、资金等资源和生产要素整合，向龙头企业、生产经营大户、专业化合作社（协会）集中，提高农牧业规模化、集约化、标准化、科技化发展水平。面向市场需求，加大呼伦贝尔肉牛、肉羊、马铃薯、黑木耳的品牌推广力度，加快阜丰玉米、合适佳芥花油等品牌建设。配套抓好包装、仓储、物流等环节，提高产品附加值。精准对接消费市场，选择部分大中城市建立呼伦贝尔代表性物产综合体验店。推动传统模式与“互联网+”模式并举，大力提高营销水平。

创新农村牧区经营制度。深化农村牧区产权制度改革，推行所有权、承包权、经营权“三权分置”，促进经营权有序流转。积极稳妥推进多种形式的适度规模经营，着力培育专业大户、家庭农牧场、农牧民专业合作社、农牧业企业等新型经营主体。健全企业与农牧民紧密型利益联结机制、技术服务体系、抗灾防灾保险体系和农畜产品质量安全可追溯体系。鼓励引导金融资本、民间资本进入农牧业。推动土地、草牧场承包经营权入股、信贷抵押等制度创新。

注重发展林业经济。利用“互联网+”探索建立森林管护新模式，加大森林抚育力度。推进红花尔基、成吉思汗大型苗圃基地和国家储备林基地建设，创造森林碳汇。大力发展林下经济，培育壮大蘑菇、木耳、蓝莓、榛子及獭兔、貂、狐等特色种养业，不断提高林区职工收入水平。

（五）以全域旅游为引擎，提升现代服务业

打造国际化高端旅游目的地。围绕原生态、国际化、全域游发展思路，大力实施全域旅游、四季旅游和“旅游+”战略。充分考虑生态环境保护需要和自然资源承载能力，科学编制全域旅游总体规划和系列专项规划。加快推进国家级旅游业改革创新先行区工作，发挥好旅游部门综合协调作用，创新旅游发展体制机制。搭建好呼伦贝尔文化旅游投资（集团）公司等投融资平台，加大旅游投入。加快蒙古之源·蒙兀室韦民族文化园、拓跋鲜卑历史文化园、柴河、白音哈达等重点景区建设，以创建北部原始林区国家公园为载体，打造泛北极（驯鹿）文化旅游景区。适度建设农区特色村落、牧区庄园牧场、林区天然氧吧、垦区特色风光等体验式精品旅游项目。在国家、自治区主流媒体和俄蒙地方台做好高层次品牌形象推介。坚决清理“小散乱”景区景点。完善旅游标识、旅游驿站、旅游厕所、救援救助中心等服务设施。加大旅游执法管理力度，规范旅游市场，营造良好的旅游消费环境。

促进文化与旅游深度融合。发挥民族、民俗等特色文化资源优势，推动文化与旅游互动发展。抓好演艺场馆建设和文化剧目编排，推出呼伦贝尔四季旅游文化演艺产品。编撰《呼伦贝尔故事》系列丛书，打造有地方代表性的文化精品，为旅游产业发展增添文化生产力。

推进新兴服务业发展。着力提升物流业发展水平，努力降低物流成本。加强国家冷链物流示范市和滨洲铁路沿线城市物流带建设，抓好海拉尔区、阿荣旗、扎兰屯市、大杨树镇等城镇物流园区各项工作。加快电子商务、金融、保险、家政、快递、健康养老等现代服务业发展。

（六）以中心城区建设为重点，推动城乡协调发展

优先发展中心城区。完善市区共建机制，启动呼伦贝尔城市总体规划评估和修编工作，对中心城区重点区域进行高品位城市设计。支持海拉尔区打造国际化“草原音乐名城”、 “冰雪运动名城”、“文化旅游名城”，将呼伦贝尔大剧院、群众艺术馆、市政府礼堂改扩建为金色大厅、银色大厅、“往日时光”音乐厅，建设草原文化国际交流中心、国际会议展览中心和冰雪运动中心。积极开展城市修补和生态修复，规划建设海拉尔湿地公园，做好辉图哈日诺尔等水体保护和东山等山体修复，努力改善中心城区的生态环境。按照城市建设发展需要，加快国家园林城市创建进程。推动游园广场、环卫设施、停车场等市政公共服务设施建设。改革体制机制、加强运营管理，进一步推进中心城区公共交通一体化发展。实施中心城区管理综合整治，规范小区物业服务，改善城市环境，提升城市形象。

统筹城乡发展。加快中心镇、重点镇产业发展和人口集聚，抓好扎兰屯市和大杨树镇国家新型城镇化综合试点建设。推进全国特色景观旅游名镇和自治区美丽宜居小镇、美丽宜居村庄创建工作。围绕建设美丽乡村，继续做好农村牧区人居环境综合整治工作，统筹推进农区、牧区、林区、垦区建设，带动基础设施、公共服务向村屯、嘎查、场队延伸覆盖。

完善基础设施。加快构建铁路网、公路网、航空网、市政网、水利网、能源网、信息通信网七大网络体系。铁路投资15亿元，确保滨洲铁路电气化改造项目竣工通车，加快阿荣旗—莫旗铁路项目建设，争取齐齐哈尔—海拉尔—满洲里客运专线列入国家“十三五”铁路发展规划，全力推进海拉尔—满洲里动车项目。公路投资100亿元，加快海拉尔—满洲里、扎兰屯—阿荣旗、尼尔基—大杨树等高等级公路建设，同时加快林区公路的规划与建设工作。民航投资2.6亿元，确保新右旗、阿荣旗、莫旗通用机场实现通航，海拉尔东山机场扩建项目如期投入运营。市政基础设施投资53.3亿元，统筹推进城市供热、供水、燃气、防洪排涝和污水垃圾处理等181个基础设施项目建设及改造。电网投资22.5亿元，加快海北—岭东500千伏输变电工程建设，确保伊敏—兴安—乌兰浩特500千伏输变电工程、滨洲铁路电气化改造项目供电工程竣工投运，争取呼伦贝尔—河北电力外送通道项目落地。水利投资25亿元，确保Z866工程、晓奇子水利枢纽工程开工，加快推进扎敦水利枢纽、甘河提水灌区等项目建设。信息通信网建设投资16亿元，实施“宽带呼伦贝尔”工程，深入推进“三网融合”。

（七）以增强发展活力为目标，深化改革开放

聚焦问题抓改革。承接好国家、自治区改革任务。深化“放管服”改革，加强事中、事后监管，着力降低制度性交易成本，激发市场活力和社会创造力。加快商事制度改革步伐，充分发挥涉企信用信息归集公示系统的作用。完成满洲里行政审批制度集中改革。强化国资监管，完善现代企业制度，深化呼伦贝尔农垦集团等国有企业改革，提高呼伦贝尔城投、林投、交投、水投等投融资主体主业发展能力。深化财税体制改革。运用好PPP模式，大力发展混合所有制经济，鼓励支持非公企业、社会资本进入基础设施和公共事业等领域，增强非公有制经济活力。启动农村牧区集体资产清查工作，深化农村牧区集体产权制度改革。推动文化、人才发展体制机制和民生领域改革。推进城市管理综合执法体制改革如期完成。

全面提高开放水平。主动融入“一带一路”战略，扩大向北开放。深入研究国家支持沿边重点地区开发开放若干政策，落实好自治区支持呼伦贝尔中俄蒙合作先导区建设的若干政策，细化责任、明确分工，确保各项政策落地并取得实效。深化海拉尔·赤塔·乔巴山金三角自由贸易区研究。提升满洲里国家重点开发开放试验区龙头带动能力，发挥满洲里综合保税区作用，完善满洲里互市贸易区配套功能。统筹规划、合理确定口岸功能定位。争取启动额尔古纳边境经济合作区建设，额布都格—巴彦呼舒中蒙跨境经济合作区取得实质性进展。继续推进额布都格、阿日哈沙特互市贸易区报批工作。加快互联互通建设，抓好口岸公路、铁路、桥梁等项目建设前期工作。强化对内协作，抓住国家实施新一轮东北振兴战略机遇，在基础设施、民生、生态、能源供应等方面加强同东北经济区的合作。加快推进海拉尔·满洲里·阿尔山经济合作区建设。完善与沿海发达地区的交流合作机制，积极承接产业转移。

（八）以增进人民福祉为根本，提高社会建设水平

实施重点民生工程。坚决打赢脱贫攻坚战，整合扶贫资源，拓宽融资渠道，推动特色产业、教育医疗和易地搬迁等扶贫项目，年内脱贫1.9万人。实施创业就业工程，加快推进各旗市区创业园、孵化基地建设，以创业带动就业，重点解决好高校毕业生、零就业家庭和退役军人等人群就业。抓好百姓安居工程，投资100亿元,开工各类保障性安居工程46692套。加快规划中心城区第二水源地，完成城镇集中式水源地保护区基础设施建设及综合治理。提高社会保障能力，完善临时救助和大病救助制度，推进全民参保计划。适度提高企业退休人员养老金、城乡低保和“五保”供养标准。完善社会化养老服务体系，提高床位补贴标准。

全面发展社会事业。深入实施创新驱动发展战略，加大对科技创新的投入力度，落实好《呼伦贝尔市人民政府关于促进科技创新若干意见》。强化企业创新主体地位，支持行业领军企业建设高水平研发机构。发挥好院士专家工作站作用，提升科技服务对科技创新和产业发展的支撑能力。继续做好国家义务教育发展基本均衡县创建工作。优先发展民族教育，大力加强职业教育，支持呼伦贝尔学院转型发展。积极推进与吉林大学等区外高校的合作。加快社会公共文化服务体系建设，推动金长城和中东铁路遗址保护工程。深化医药卫生体制改革，推进分级诊疗制度建设，强化医疗卫生三级网络。争取在蒙中医药研究方面有所突破。完善计划生育服务管理，提高出生人口素质。加快食品药品安全监管体系和应急处置能力建设，保障人民群众饮食、用药安全。加大各类体育场馆建设力度，承办好自治区第九届少数民族传统体育运动会，有序推进海拉尔区、牙克石市、扎兰屯市第十四届全国冬季运动会场馆建设及改造工作。发展民族、冰雪等传统优势体育项目，推进足球运动改革，提高体育竞技水平。促进全民健身与全民健康融合发展，打造“健康呼伦贝尔”。做好第三次全国农业普查。提升防灾减灾和气象预警能力。做好残疾人、老龄人群、妇女儿童权益保护工作，支持红十字和公益慈善事业发展。统筹抓好人防、地震和档案史志等工作。

稳步推进社会治理。认真贯彻执行党的民族政策，全力创建全国民族团结进步示范市。做好创建全国文明城各项工作，以创促建，努力提高全社会文明程度。构建农村牧区和社区服务体系，提高社会服务水平。深入开展社会矛盾大排查、大化解和积案化解百日攻坚专项行动，压实信访责任，强化源头治理，妥善解决群众合理诉求。完善立体化社会治安防控体系，依法严厉打击各类违法犯罪活动，维护良好的社会治安环境，切实增强群众的安全感和满意度。高度重视安全生产工作，认真落实“党政同责、一岗双责”制度，坚决遏制重特大事故发生。进一步强化责任意识，提高应急保障能力，做好森林、草原防火工作。重视网络舆情监管和舆情引导。加大信息化管边控边力度，提升边境综合防卫能力。巩固军政军民关系，维护民族团结、边疆安宁、和谐稳定的良好局面。

（九）以执政为民为宗旨，加强政府自身建设

坚持依法行政。围绕市委的重大决策和工作部署，严格执行“三重一大”决策程序，不断提高行政决策民主化、科学化、法治化水平。完善行政复议及行政诉讼应诉制度，加强规范性文件审查和备案审查。认真开展法律援助活动，实现法律顾问制度全覆盖。坚持依法监督权力，自觉接受人大法律监督、政协民主监督和社会公众监督，认真办理好人大代表议案、建议和政协委员提案，广泛听取各民主党派、工商联、无党派人士意见，支持工会、共青团、妇联等人民团体发挥作用。

坚持科学施政。加强政治理论、法律知识和业务知识学习，注重调查研究，准确把握市情。强化改革意识，创新工作机制和工作方法，摒弃束缚发展的惯性思维，切实提高各级领导干部研究新情况、解决新问题、总结新经验的能力，使制定的各项政策措施更加符合呼伦贝尔实际。

坚持高效执政。强化执政为民意识，转变工作作风，提高地区、部门协同协作水平，形成工作合力。加快推进“互联网+政务服务”工作，打造便捷高效的政务服务体系，大力提高政府执行力和行政效能。建立干部容错纠错机制，鼓励干部积极作为，克服“慵懒散”，严厉问责“不作为”、“慢作为”、“乱作为”等行为。政府要谋大事、顾大局，围绕中心工作找方位、定坐标，盯住重点项目、重大事项，加强工作调度和督促检查，切实提高抓落实的能力。

坚持清廉从政。牢固树立“政治意识、大局意识、核心意识、看齐意识”。严格落实《关于新形势下党内政治生活的若干准则》和《中国共产党党内监督条例》。进一步强化党风廉政建设主体责任，认真落实好“一岗双责”制度。加强行政监察和审计监督，加大对重点领域、关键岗位的监管力度，保持惩治腐败高压态势，以无禁区、零容忍的态度营造风清气正的良好环境。

各位代表！新目标鼓舞人心，新任务光荣艰巨，新征程催人奋进！让我们紧密团结在以习近平同志为核心的党中央周围，在自治区党委、政府和市委的坚强领导下，守望相助，团结奋斗，一往无前，全力开创呼伦贝尔市改革发展新局面，为实现美丽发展、争进前列，全面建成小康社会，为把祖国北疆这道风景线打造得更加亮丽而努力奋斗！
